# Application of the Random Forest Algorithm for Accurate Bipolar Disorder Classification

**DOI:** 10.3390/life15030394

**Published:** 2025-03-03

**Authors:** Miguel Suárez, Ana M. Torres, Pilar Blasco-Segura, Jorge Mateo

**Affiliations:** 1 Virgen de la Luz Hospital, 16002 Cuenca, Spain; 2 Medical Analysis Expert Group, Institute of Technology, University of Castilla-La Mancha, 13001 Cuenca, Spain; 3 Instituto de Investigación Sanitaria de Castilla-La Mancha (IDISCAM), 45071 Toledo, Spain; 4 Department of Pharmacy, General University Hospital, 46014 Valencia, Spain

**Keywords:** bipolar disorder, machine learning, random forest, artificial intelligence, classification

## Abstract

Bipolar disorder (BD) is a complex psychiatric condition characterized by alternating episodes of mania and depression, posing significant challenges for accurate and timely diagnosis. This study explores the use of the Random Forest (RF) algorithm as a machine learning approach to classify patients with BD and healthy controls based on electroencephalogram (EEG) data. A total of 330 participants, including euthymic BD patients and healthy controls, were analyzed. EEG recordings were processed to extract key features, including power in frequency bands and complexity metrics such as the Hurst Exponent, which measures the persistence or randomness of a time series, and the Higuchi’s Fractal Dimension, which is used to quantify the irregularity of brain signals. The RF model demonstrated robust performance, achieving an average accuracy of 93.41%, with recall and specificity exceeding 93%. These results highlight the algorithm’s capacity to handle complex, noisy datasets while identifying key features relevant for classification. Importantly, the model provided interpretable insights into the physiological markers associated with BD, reinforcing the clinical value of EEG as a diagnostic tool. The findings suggest that RF is a reliable and accessible method for supporting the diagnosis of BD, complementing traditional clinical practices. Its ability to reduce diagnostic delays, improve classification accuracy, and optimize resource allocation make it a promising tool for integrating artificial intelligence into psychiatric care. This study represents a significant step toward precision psychiatry, leveraging technology to improve the understanding and management of complex mental health disorders.

## 1. Introduction

Bipolar disorder (BD) is a complex psychiatric illness that affects millions of people worldwide and is associated with drastic changes in mood, energy, and behavior. These changes can range from episodes of extreme euphoria, known as mania, to prolonged periods of deep depression, significantly impacting patients’ quality of life as well as their social and family environments. Recent studies estimate that between 1% and 3% of the global population suffers from bipolar disorder [1,2], making it a highly relevant public health issue. Despite advances in research and diagnostic methods, bipolar disorder remains a challenge for healthcare professionals due to the diversity in its clinical presentation and the overlap of its symptoms with other mood disorders, such as major depressive disorder [3,4].

The diagnosis of bipolar disorder is a complex process influenced by multiple clinical and neurobiological factors. In addition to the core symptoms of the disorder, temperamental traits have been shown to play a key role in identifying and differentiating BD from other psychiatric disorders. Recent studies, such as that of Favaretto et al. [5], have explored the relationship between affective temperaments and mood disorders, highlighting their potential to improve diagnostic accuracy [1]. In particular, certain temperamental profiles, such as cyclothymia and hyperthymia, have been linked to greater susceptibility to BD, suggesting that integrating these dimensions with machine learning-based tools could enhance current diagnostic approaches.

The diagnosis of bipolar disorder remains a significant challenge in clinical practice due to multiple factors. First, it relies heavily on the subjective evaluation of symptoms through clinical interviews, which can lead to variations in interpretations among different professionals. Second, there is symptomatic overlap with other psychiatric disorders, such as major depressive disorder and borderline personality disorder, complicating precise differentiation. Additionally, studies have reported that the average time to reach a definitive BD diagnosis can exceed 10 years, during which patients may receive inappropriate treatments that exacerbate the condition [1,2,3]. These limitations highlight the need for objective, data-driven tools that complement traditional clinical evaluation.

The early and accurate diagnosis of bipolar disorder is crucial for improving therapeutic outcomes and preventing associated complications such as suicide, medical comorbidities, and functional impairment [1,6]. However, the current diagnostic process still heavily relies on clinical observation and the subjective interpretation of symptoms by specialists, which carries a considerable risk of errors and delays in diagnosis [7]. These limitations underscore the need to develop more objective, data-driven tools that can complement clinical evaluation and provide a more precise and efficient approach to diagnosing bipolar disorder. Studies have analyzed the similarities and differences in the classification of bipolar disorders according to the DSM-5 and the beta version of the ICD-11, highlighting the incorporation of dimensional parameters for symptom assessment and the inclusion of new course specifiers, such as mixed features [8]. A review emphasized the latest advances in the diagnosis and treatment of bipolar disorder, stressing the importance of precision psychiatry and the need for thorough evaluation of patients with depressive symptoms to identify manic or hypomanic episodes [9]. Another study provided a comprehensive review of bipolar affective disorder, addressing its prevalence, clinical presentation, and treatment options, while emphasizing the importance of tailoring treatment to each patient [10]. Research has also explored the classification, epidemiology, and etiopathogenesis of bipolar disorder, offering an integrated perspective on the disease and its clinical implications [11].

In this context, machine learning (ML) approaches have emerged as promising alternatives to improve the accuracy and speed of BD detection [12,13,14]. ML enables the automated analysis of large volumes of biomedical data, identifying subtle patterns that may be difficult to detect using traditional methods. In particular, the application of ML in electroencephalographic (EEG) signal processing has demonstrated its ability to reveal neurophysiological alterations associated with BD, providing an objective biomarker-based approach for patient classification [7,8,9]. This study explores the use of the Random Forest (RF) algorithm for the automated classification of BD patients and healthy controls, aiming to contribute to the development of complementary tools that optimize the diagnostic process in precision psychiatry.

In recent decades, data-driven medicine has experienced significant growth, driven by the increasing availability of large clinical databases and the development of artificial intelligence tools such as machine learning. Machine learning algorithms have emerged as promising tools for improving diagnostic accuracy by analyzing large volumes of clinical, neurophysiological, and behavioral data [15,16,17,18,19,20]. One study proposed a deep learning method utilizing actigraphy and electrodermal activity data obtained from wrist-worn devices to differentiate between manic and euthymic states in bipolar patients [21]. Another study employed a machine learning model based on mathematical signatures to differentiate between bipolar disorder and borderline personality disorder [22,23]. Pettorruso et al. developed a machine learning-based model to predict the response to intranasal esketamine in patients with treatment-resistant depression, achieving an accurate classification of patients who responded favorably to the treatment [24]. Similarly, Distefano et al. applied AI models to the analysis of functional magnetic resonance imaging (fMRI) data to improve the detection of schizophrenia, highlighting the potential of these tools in precision psychiatry [25].

Additionally, a system using one-dimensional convolutional neural networks has been applied to analyze intrinsic connectivity patterns in resting-state functional magnetic resonance imaging (fMRI) data [26]. Various machine learning algorithms such as Random Forest (RF) [27,28], Support Vector Machines (SVM) [29,30,31,32], k-Nearest Neighbors (KNN) [33,34,35], decision trees (DT) [36,37,38], and Gaussian Naïve Bayes (GNB) [39,40] are being used to process medical data [41,42,43], achieving high accuracy rates. These techniques have demonstrated not only their ability to identify complex patterns associated with bipolar disorder but also to provide more objective and consistent interpretations compared to traditional methods [44]. Within this context, the RF model has gained popularity due to its robustness, flexibility, and ability to handle noisy and nonlinear data. This method, based on an ensemble of decision trees, has been widely applied in various medical domains, showing outstanding performance in disease classification, clinical outcome prediction, and the selection of relevant features in biomedical studies [28,44,45].

The objective of the present study is to develop a system capable of predicting patients with bipolar disorder using the RF algorithm based on a patient database through the analysis of their electroencephalogram (EEG) data. This approach aims to address some of the main challenges associated with traditional diagnosis, such as subjectivity and delays in identifying the disorder, and to evaluate the efficacy of a predictive model based on EEG recordings. Additionally, the study seeks to identify the most influential features in the classification of patients, providing a clearer perspective on the factors associated with the development of bipolar disorder. The implementation of this model could have a significant impact on clinical practice by offering a complementary tool for the early and accurate diagnosis of bipolar disorder.

## 2. Materials and Methods

### 2.1. Materials

In this study, real EEG recordings were used to develop a predictive system for identifying patients with bipolar disorder. We analyzed the individual results of 330 participants, 120 of whom were patients with bipolar disorder (BD) and 210 were healthy controls. In response to the observations, a diagnosis was made according to DSM-IV criteria, and patients received treatment in the Severe Mental Disorders Program (SMD-Cu) at the Department of Psychiatry of the Hospital. Each diagnosis was confirmed through the Structured Clinical Interview for DSM-IV (SCID-I). At the time of evaluation, all patients met the euthymia criteria applied in our previous studies (scores < 7 on the Hamilton Depression Rating Scale [HDRS] and <6 on the Young Mania Rating Scale [YMRS], at least three months prior to the study).

EEG data were recorded using a 32-channel BrainVision Recorder. Brain Products GmbH. Gilching (Germany) system with a sampling rate of 500 Hz. Electrodes were placed according to the International 10–20 System, and impedance was maintained below 10 kΩ to minimize signal distortion. To reduce the influence of spurious signals, several techniques were applied. A band-pass filter (0.5–45 Hz) based on a fourth-order Butterworth filter was used to eliminate low-frequency noise, such as movement artifacts and baseline fluctuations, as well as high-frequency components caused by electronic interference and muscular noise. Additionally, the Independent Component Analysis (ICA) method was implemented to correct muscular and ocular artifacts, allowing the identification and removal of components related to eye movements, such as blinking and saccadic movements, as well as muscle activity. Components associated with these artifacts were visually inspected and removed before further analysis. A manual inspection of channels was also performed to detect extreme values or persistent noises. If a defective channel was found, its signal would be replaced using weighted interpolation from neighboring electrodes.

To ensure data uniformity before analysis, segmentation and normalization processes were applied. Signals were divided into 2-s segments with a 50% overlap to maximize data availability while preserving information. Subsequently, each EEG segment was normalized using the Z-score technique, adjusting the mean and standard deviation for each channel. This step ensured that the extracted features had a homogeneous scale and reduced the influence of individual variations in the signal.

From the preprocessed EEG signals, different feature sets were calculated. In the temporal domain, descriptive statistics such as mean, variance, kurtosis, and skewness were extracted to capture the distribution of brain electrical activity. In the frequency domain, the Fast Fourier Transform (FFT) was applied to decompose the signal into frequency bands, including delta, theta, alpha, beta, and gamma, allowing for an evaluation of the relative power in each band. In the nonlinear domain, complexity metrics such as Higuchi’s Fractal Dimension, the Hurst Exponent, and the Lyapunov Exponent were computed, providing information on the irregularity and chaotic dynamics of brain signals.

The study was approved by the Clinical Research Ethics Committee of the Cuenca Health Area, and all participants provided informed consent after receiving a detailed explanation of the procedures involved. The study was conducted in accordance with the ethical principles outlined in the Declaration of Helsinki for medical research involving human subjects, ensuring the rights, welfare, and dignity of the participants were respected.

### 2.2. Model Development

In this study, the RF algorithm was employed to address the classification task of distinguishing between patients with bipolar disorder and healthy controls. RF is an ensemble model based on decision trees, designed to handle complex and nonlinear data with high robustness against noisy and imbalanced datasets [46]. This algorithm has proven particularly effective in biomedical problems due to its ability to identify relevant features and its flexibility in multivariate scenarios [33]. The RF model was trained using features extracted from EEG signals. A total of 100 decision trees were configured in the model, and the number of features considered at each split was set to the square root of the total number of features, following standard recommendations to optimize the balance between bias and variance [28,44,45].

Feature extraction represents a fundamental step in EEG signal analysis, as it enables the derivation of relevant attributes that encapsulate critical information about the underlying neurophysiological patterns in bipolar disorder. In this study, advanced techniques were implemented to extract features from EEG signals in the temporal, frequency, and nonlinear domains, with a focus on maximizing their clinical relevance and their ability to enhance the performance of predictive models.

In the temporal domain, statistical metrics describing the basic properties of EEG signals were calculated. These included the mean, variance, kurtosis, and skewness, which characterize the amplitude distribution of the signals. For instance, kurtosis is particularly useful for identifying extreme events or peaks in EEG signals, which may be associated with pathological brain states. These metrics provide an initial framework for understanding general variations in brain electrical activity between patients with bipolar disorder and healthy controls [28,43,44,45].

In the frequency domain, the Fast Fourier Transform (FFT) was used to decompose EEG signals and analyze spectral power in the delta (0.5–4 Hz), theta (4–8 Hz), alpha (8–13 Hz), beta (13–30 Hz), and gamma (>30 Hz) bands. Each of these bands is associated with specific brain functions, such as alertness (beta), relaxation (alpha), and emotional processes (theta). Previous studies have shown that alterations in these bands may serve as potential biomarkers in psychiatric disorders, including bipolar disorder [47,48]. For example, a decrease in alpha band power and an increase in beta activity have been reported during manic episodes.

In the nonlinear domain, advanced metrics were applied to capture the complex and chaotic dynamics of EEG signals, which are often not evident through linear analysis. Higuchi’s Fractal Dimension, the Lyapunov Exponent, and the Hurst Exponent were calculated. Higuchi’s Fractal Dimension measures the irregularity and geometric complexity of the signals, while the Lyapunov Exponent quantifies sensitivity to initial conditions, providing a measure of chaos in the system [47,48]. The Hurst Exponent, on the other hand, evaluates the signal’s tendency to maintain persistent or antipersistent behavior over time, making it useful for detecting dynamic patterns in pathological conditions [47].

To optimize the feature set and prevent overfitting in machine learning models, a feature selection procedure based on the relative importance of attributes was implemented. Mutual information-based selection was used to identify the most discriminative features, reducing the dataset’s dimensionality and ensuring that only the most relevant variables were included. This approach not only enhances the predictive power of the models but also facilitates better interpretation of the results by highlighting the most significant biomarkers associated with bipolar disorder [44]. The combination of these multidimensional approaches ensures that the extracted features not only capture essential information about brain activity but are also robust to noise and individual variations. These features represent an optimal input set for machine learning algorithms such as RF [28,44,45,46], SVM [29,30,31,32], KNN [33,34,35], DT [36,37,38], and GNB [39,40] which require clean and representative data to achieve accurate classification.

To evaluate the model’s performance, a 5-fold cross-validation scheme was used. This approach ensured that each dataset was used for both training and testing, minimizing the risk of overfitting and providing a more reliable assessment of the model [43]. The dataset was divided into two subsets, with 70% allocated for training and 30% for testing, ensuring the independence of patient groups between the sets. Key performance metrics, including Accuracy, Recall, Specificity, F1 Score, and the area under the ROC curve (AUC), were calculated to evaluate the model’s effectiveness in classifying patients.

## 3. Results

In this study, the RF algorithm was used to address the classification task of distinguishing between patients with bipolar disorder and healthy control subjects, based on processed EEG data and extracted features. This method, based on an ensemble of decision trees, stands out for its ability to handle complex and nonlinear data, as well as its robustness against data noise. To evaluate the model’s performance, a 5-fold cross-validation scheme was implemented, ensuring independence between training and testing data and minimizing the risk of overfitting.

The proposed RF system was compared with four classification algorithms: SVM, DT, GNB, KNN, and RF, using a set of evaluation metrics to measure their performance: Accuracy, Matthews Correlation Coefficient (MCC), F1 Score, Precision, DYI, Recall, Specificity, Kappa, and AUC. The results obtained are presented in Table 1 and Table 2.

The results show that the RF algorithm significantly outperformed the other evaluated methods, achieving an Accuracy of 93.41%, an AUC of 0.93, and the highest values for Recall (93.51%), Specificity (93.30%), F_1_ Score (93.13%), and MCC (82.99%). In comparison, the second-best classifier, KNN, achieved an Accuracy of 85.44% and an AUC of 0.85, while SVM and DT algorithms showed moderate performance, with an Accuracy of 83.65% and 82.35%, respectively. On the other hand, GNB had the lowest performance, with an Accuracy of 74.72% and an AUC of 0.75, highlighting its limitations in handling the complexity of the data. The combination of Recall and Specificity offered by RF surpasses that of other methods, underscoring its robustness in complex classification scenarios. Furthermore, the high Kappa (83.27) and Precision (93.75%) indicate its ability to minimize both false negatives and false positives. The proposed system also achieves an AUC value close to 0.93, exceeding KNN by 8%, with the other algorithms showing less precise values. These results position the proposed RF system as a reliable, robust, and superior model for classifying patients with bipolar disorder.

**Figure 1:**
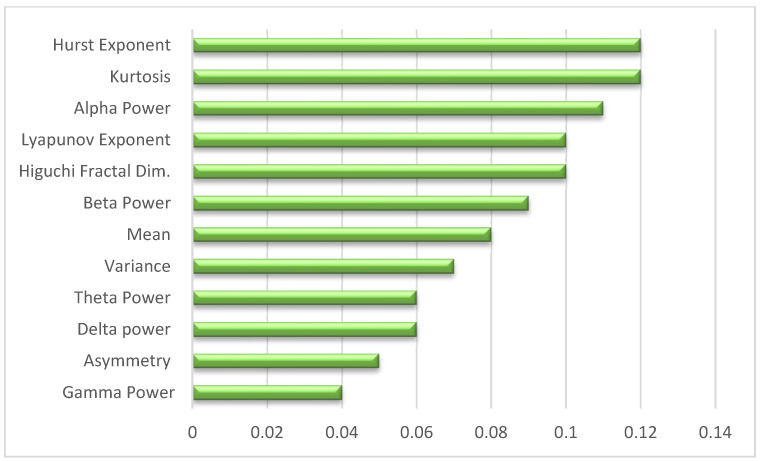
The figure represents the importance of the extracted features in the classification of patients.

The classification analysis of patients with bipolar disorder based on EEG signals revealed that features extracted from the temporal, frequency, and nonlinear domains significantly contribute to the model. As shown in Figure 1, in the temporal domain, kurtosis emerged as the most important metric (12%), followed by mean, variance, and skewness, which characterized the basic statistical properties of the signals. In the frequency domain, the power in the alpha (11%) and beta (9%) bands showed high relevance associated with relaxation and cognitive processing, respectively, while the delta, theta, and gamma bands provided complementary information. Nonlinear metrics, such as Higuchi’s Fractal Dimension (10%) and the Hurst (12%) and Lyapunov (10%) exponents, were crucial for capturing the complexity and chaotic dynamics of EEG signals, offering unique insights into pathological alterations. These findings highlight the need for a multidimensional approach that combines features from different domains to capture the complexity of bipolar disorder.

**Figure 2:**
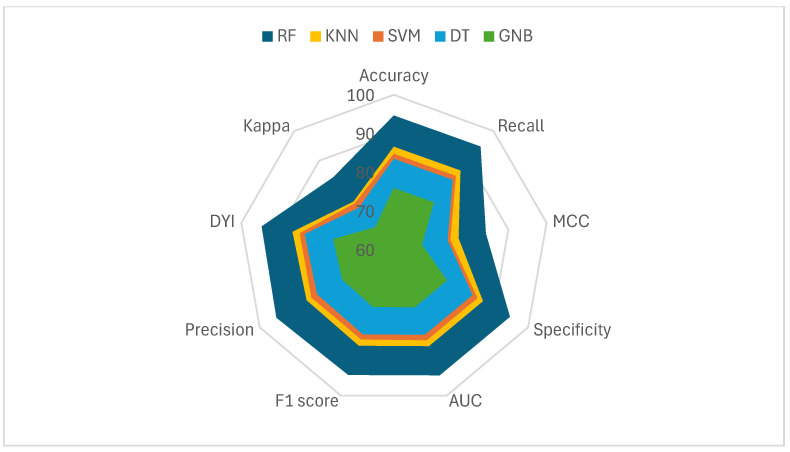

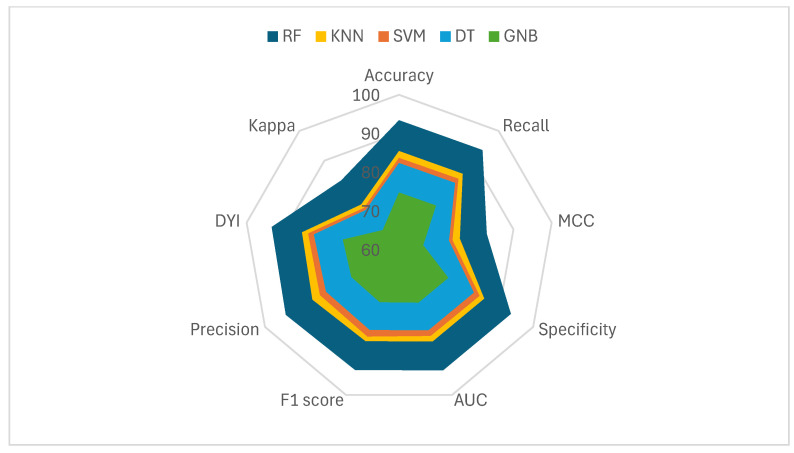
The figure represents the radar plots of the different algorithms studied. The upper figure shows the training results, while the lower figure displays the test results.

As shown in Figure 2, the training subsets of the model and the test subset exhibit high scores across all metrics, although they are slightly lower in the test subset. This consistency is due to the algorithm achieving an optimal level of training without incurring overfitting or underfitting. As observed in Figure 1, the RF model covers a larger area compared to the other evaluated methods, demonstrating a well-balanced model with high generalization capacity and the ability to provide accurate results with new data.

**Figure 3:**
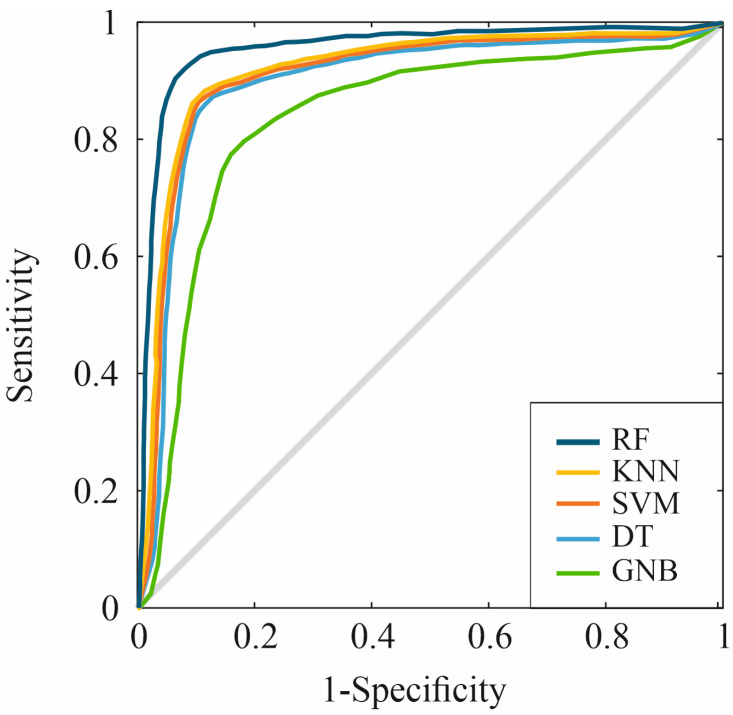
The figure represents the ROC curve for different machine learning algorithms.

Additionally, the ROC curve was generated to represent sensitivity and specificity measures for each threshold value, aiming to evaluate the classification capabilities of the different machine learning algorithms. The results, presented in Figure 3, once again show that the proposed RF-based system covers a larger area, indicating superior predictive accuracy.

Compared to other evaluated classification methods, RF demonstrated competitive performance. Its ease of interpretation and scalability make RF an attractive tool for clinical applications, where transparency in the decision-making process is crucial.

The results obtained support the use of RF as an effective approach for the automated classification of patients with bipolar disorder, showing robust performance in terms of accuracy, sensitivity, and specificity. This model not only enables faster and more objective diagnoses but also provides valuable insights into the most relevant features for identifying this condition, which could inform future developments in the field of computational psychiatry. These findings highlight the potential of machine learning-based methods to complement and enhance current diagnostic practices for complex disorders like bipolar disorder.

## 4. Discussion

The early and accurate detection of bipolar disorder is crucial due to the significant impact this condition has on patients, their families, and society at large. This disorder, characterized by extreme mood swings oscillating between manic and depressive episodes, affects between 2% and 3% of the global population and is associated with high rates of comorbidity, disability, and suicide risk [1]. Without an accurate diagnosis, many patients receive incorrect treatments, prolonging their suffering and increasing the economic and social costs associated with the disease [49]. For these reasons, the timely identification of bipolar disorder should be a priority for healthcare systems.

One of the main challenges in detecting bipolar disorder is the reliance on clinical interviews and the subjective interpretation of symptoms, which leads to variability in diagnoses. The overlap with other mood disorders and borderline personality disorder increases the difficulty in accurately identifying BD, often resulting in misdiagnoses and delays in the implementation of appropriate treatments. These factors have driven the search for complementary methods that integrate neurophysiological biomarkers and automated approaches to improve diagnostic accuracy. In this study, we demonstrated that the Random Forest (RF)-based model applied to EEG provides a robust tool for BD classification with an accuracy of 93.41%, outperforming other algorithms such as SVM and KNN in key metrics. However, although this EEG-based approach enhances diagnostic objectivity, its clinical implementation requires further validation, and its combination with clinical data could further enhance its utility in hospital settings. For EEG data classification, multiple machine learning algorithms were evaluated, including Random Forest (RF), Support Vector Machines (SVM), k-Nearest Neighbors (KNN), decision trees (DT), and Gaussian Naïve Bayes (GNB). After a comparative analysis, RF was selected as the final model due to its superior performance in terms of accuracy, robustness, and interpretability. EEG signals are highly variable and prone to noise, which poses challenges for classification. RF is particularly well-suited for handling noisy and high-dimensional data, as it leverages an ensemble of decision trees, reducing overfitting and ensuring stable generalization to new datasets. Additionally, RF can capture nonlinear relationships in EEG signals without requiring complex transformations, unlike SVM with a linear kernel or GNB, which rely on specific assumptions about data distribution.

Recent studies have explored deep learning (DL) techniques, such as Convolutional Neural Networks (CNNs) and Recurrent Neural Networks (RNNs), for EEG-based BD classification. While these models have demonstrated strong performance in various classification tasks, they require large amounts of labeled data, which can be difficult to obtain in clinical settings due to data collection challenges and ethical constraints. RF, on the other hand, is more data-efficient, performing well even with limited sample sizes, making it a more practical option for real-world medical applications. Furthermore, RF offers greater interpretability, allowing for the identification of key EEG biomarkers, such as power in the alpha and beta bands and complexity metrics like Higuchi’s Fractal Dimension and the Hurst Exponent, whereas deep learning models often act as black-box classifiers, limiting insight into their decision-making process.

Additionally, RF exhibits superior robustness to noise and computational efficiency compared to deep learning models. EEG signals are inherently noisy, and CNNs, in particular, are highly sensitive to artifacts, often requiring extensive preprocessing. Without proper augmentation and hyperparameter tuning, CNNs and RNNs may struggle to generalize well. In contrast, RF’s ensemble-based approach mitigates overfitting and ensures stable performance across varying data conditions. Moreover, RF does not require specialized hardware such as GPUs, unlike deep learning models which demand high computational resources, making RF a more accessible and scalable solution for EEG-based BD classification. While deep learning models have achieved promising results in EEG classification, our comparative analysis confirmed that RF outperformed alternative machine learning models and remained competitive with deep learning approaches, achieving a 93.41% accuracy while maintaining interpretability, computational efficiency, and robustness to noise. Given these advantages, RF was determined to be the most suitable model for this study.

Although this study focused on EEG data analysis, recent research has highlighted the relevance of temperamental factors in BD assessment [5]. The integration of these traits with machine learning approaches could yield more precise hybrid models, combining neurophysiological biomarkers with behavioral and temperamental characteristics. Future research directions should explore how the combination of these dimensions can improve the differentiation of BD from other psychiatric disorders.

The heterogeneous nature of bipolar disorder and its symptomatic overlap with other mood disorders, such as unipolar depression, complicates its clinical diagnosis. Indeed, previous studies have shown that the average time to reach an accurate diagnosis can exceed 10 years, during which patients remain at risk of developing severe complications, such as psychotic episodes, substance abuse, and functional impairment [50]. The introduction of artificial intelligence tools, such as the RF algorithm evaluated in this study, can play a transformative role by providing complementary methods to reduce diagnostic delays and improve detection accuracy.

The analysis of EEG signals using machine learning techniques has significantly advanced the classification of patients with bipolar disorder. In the temporal domain, metrics such as mean, variance, kurtosis, and skewness have proven useful for characterizing the statistical properties of EEG signals, providing essential information about amplitude distribution and the presence of extreme events. These metrics have been fundamental in recent studies exploring the differentiation between normal and pathological patterns in brain activity [51,52].

In the frequency domain, the FFT enables the decomposition of EEG signals to analyze spectral power in specific bands, such as delta, theta, alpha, beta, and gamma. Each band is associated with specific brain functions, and alterations in these frequencies have been identified as potential biomarkers in psychiatric disorders, including bipolar disorder. For example, studies have reported decreased alpha power and increased beta activity during manic episodes, highlighting the importance of these features for diagnosis [51,52,53,54].

The nonlinear domain provides a unique perspective by capturing the complexity and chaotic dynamics of EEG signals through metrics such as Higuchi’s Fractal Dimension, the Lyapunov Exponent, and the Hurst Exponent. These metrics allow the analysis of properties such as self-similarity and the persistent or antipersistent behavior of signals, which are particularly relevant for pathological conditions like bipolar disorder [51,52,53,54]. Combining these metrics with features from other domains has enhanced classification performance in various studies.

Machine learning algorithms have been instrumental in leveraging these features for classifying patients with bipolar disorder. Methods such as SVM, neural networks, and ensemble approaches have proven effective, achieving high accuracy in differentiating patients from healthy controls [55,56,57,58]. Integrating EEG data with other modalities, such as cognitive assessments and neuroimaging, has shown great potential for enhancing diagnostic accuracy and providing a more comprehensive understanding of the neurophysiological differences between bipolar disorder (BD) and other psychiatric conditions [57,58,59,60,61,62]. Functional neuroimaging techniques like fMRI, PET, and DTI could offer a more detailed view of neural circuits, complementing EEG data by capturing structural and functional brain alterations associated with BD. Additionally, incorporating genetic and molecular biomarkers into machine learning models could improve personalized diagnostics, allowing for a more tailored approach to identifying individuals at higher risk. Beyond biological markers, wearable sensors—such as smartwatches tracking electrodermal activity and heart rate variability—could provide real-time physiological data that help detect manic or depressive episodes. These multimodal approaches have the potential to refine predictive models, reduce misclassifications, and contribute to a more precise understanding of BD’s neurophysiological mechanisms, ultimately leading to more accurate and individualized diagnostic strategies.

Functional neuroimaging (fMRI, PET, DTI) could provide a more detailed view of neural circuits, complementing EEG data. Additionally, incorporating genetic and molecular biomarkers into machine learning models could improve personalized diagnostics. Wearable sensors, such as smartwatches tracking electrodermal activity and heart rate variability, may also offer real-time physiological data to detect manic or depressive episodes. These multimodal approaches could refine predictive models, reduce misclassifications, and contribute to a more precise understanding of BD’s neurophysiological mechanisms.

In this study, RF demonstrated strong performance in classifying patients with bipolar disorder using features derived from EEG data, achieving an accuracy of 93.41% along with high sensitivity and specificity metrics. These figures are encouraging, as they suggest that the model has the potential to reliably identify both patients and controls, reducing the margin of error associated with traditional methods based on clinical interviews and subjective observations. Furthermore, the model analysis identified relevant biomarkers, such as brain complexity metrics (the Hurst Exponent and Higuchi’s Fractal Dimension) and power in the alpha and beta frequency bands, reinforcing the utility of EEG for the objective diagnosis of bipolar disorder.

Early detection has significant clinical implications. A timely diagnosis can facilitate the initiation of more appropriate treatments, such as mood stabilizers, and reduce exposure to ineffective or harmful therapies, such as the overuse of antidepressants in bipolar patients misdiagnosed with unipolar depression [3]. Moreover, early and accurate detection enables the implementation of preventive strategies that mitigate the risk of future episodes, improve patients’ quality of life, and reduce the disease’s impact on social and occupational domains.

Detecting bipolar disorder is not only essential for improving individual patient outcomes but also for reducing the societal burden of this illness. Tools like RF represent a significant step toward more objective, accessible, and data-driven psychiatry, paving the way for transforming how complex mental disorders are understood and managed. This approach not only enhances diagnostic accuracy but also offers the potential to transform patient care by facilitating earlier diagnoses, more effective treatments, and improved quality of life.

## 5. Conclusions

This study demonstrates that the Random Forest (RF) algorithm is an effective and reliable tool for the automated classification of bipolar disorder (BD) using EEG data, achieving an accuracy of 93.41%. This suggests that artificial intelligence can complement traditional clinical practices by providing more objective, faster, and reproducible evaluations. The implementation of AI-based tools could significantly reduce delays in BD diagnosis, which currently can exceed 10 years. An early diagnosis would allow for timely initiation of appropriate treatments, reducing the risk of severe episodes, hospitalizations, and associated complications. Additionally, by improving the differentiation between BD and other mood disorders, such as major depression, it could optimize medication use and prevent the inappropriate prescription of antidepressants to bipolar patients.

This study represents a key advancement in precision psychiatry, demonstrating the potential of machine learning to enhance BD assessment and management. The integration of AI models into clinical practice could transform psychiatric diagnosis, leading to more personalized, efficient, and evidence-based patient care.

## Figures and Tables

**Table 1:** The table presents the results for Accuracy, MCC, F_1_ Score, Precision, and DYI.

	Accuracy	MCC	F_1_ Score	Precision	DYI
SVM	83.65	73.98	83.90	83.56	83.85
DT	82.35	72.95	82.11	81.77	82.31
GNB	74.72	66.30	74.50	74.19	74.69
KNN	85.44	75.93	85.19	85.84	85.34
RF	93.41	82.99	93.13	93.75	93.37

**Table 2:** Metrics for Recall, Specificity, Kappa, and AUC are summarized in the table.

	Recall	Specificity	Kappa	AUC
SVM	83.85	83.95	74.03	0.84
DT	82.45	82.26	73.32	0.82
GNB	74.81	74.63	66.52	0.75
KNN	85.54	85.34	75.18	0.85
RF	93.51	93.30	83.27	0.93

## Data Availability

The datasets employed and analyzed in the current study are accessible upon reasonable request from the corresponding author.
